# A survey on the causes of headache among pregnant women attending the emergency department (Imam Khomeini Hospital, Sari)

**Published:** 2012-11

**Authors:** Seyyed Hossein Montazer

**Affiliations:** ^a^Department of Emergency Medicine, Mazandran University of Medical Science, Mazandaran, Iran.

## Abstract

**Background:**

Delivering and growing up a healthy child is one the most common wishes of all women worldwide. Therefore, all women regularly evaluate the progress and conditions of their health during pregnancy to avoid any potential risk factors. Pregnancy itself imposes different physiological and behavioral changes in the pregnant woman and different factors influence the pregnancy conditions and outcomes. The present study aims to evaluate the reasons and causes of headache among pregnant women attending the emergency department (Imam Khomeini Hospital, Sari).

**Methods:**

This was a descriptive cross-sectional study conducted 30 pregnant women attending the emergency department because of headache during October to December 2011. Sampling method was counting all patients.The patients were assessed at the end of their treatment process using a customized questionnaire. All patients attended the department for treatment during the study period voluntarily answered the items of the questionnaire.

**Results:**

A total of 30 pregnant women complaining about persistent headaches attended the emergency department. They were assessed for the pregnancy poisoning. 19 patients experienced headache during the third trimester of pregnancy. Assay poison of pregnancy women &19 women stricken headaches in three month last of pregnancy.

**Conclusions:**

If the headaches of pregnant women in their third trimester of pregnancy are accompanied by hypertension and proteinuria, they should visit a specialist to assess the presence of pre-eclampsia. When the pre-eclampsia test is positive, the patient should be seriously treated. Proteinuria test was performed on all patients and all of them have hypertension. In a rare condition the hypertension and proteinuria occur in first days of post-delivery. The cause of high blood pressure may be the pregnancy itself which is transient, or the patients had hypertension prior to the pregnancy and not aware of their problem. In the latter case, the problem can persist after pregnancy. Long-time hypertension increases the risk of pre-eclampsia so that 25% women, who have hypertension, experienced pre-eclampsia, while only 5% pregnant women usually experience pre-eclampsia. Therefore, if the blood pressure is high in a pregnant woman without any previous history, it cannot be predicted that the hypertension either remains unchanged, increases or leads to pre-eclampsia. If the patient catches pre-eclampsia, the proteinuria test shows excess level of urine protein that leads to swelling in the arms and legs. This symptom indicates the failure of kidney function as a result of hypertension. In some cases both the mother and fetus are under serious risks. Pre-eclampsia and hypertension are among serious health threats for pregnant women and also the most common cause of mortality in pregnant women. Therefore, presence of headache in the third trimester of pregnancy should be treated seriously.

**Keywords:**

Pregnancy, Pre-eclampsia, Headache, Third trimester, Proteinuria


Volume 4, Suppl. 1 Nov 2012
Publisher: Kermanshah University of Medical Sciences
URL: http://www.jivresearch.org
Copyright: (cc) BY

**Paper No. 87**

### A survey on the causes of headache among pregnant women attending the emergency department (Imam Khomeini Hospital, Sari)

Seyyed Hossein Montazer [a,*]

[a] Department of Emergency Medicine, Mazandran University of Medical Science, Mazandaran, Iran.

### Abstract:

**Background:** Delivering and growing up a healthy child is one the most common wishes of all women worldwide. Therefore, all women regularly evaluate the progress and conditions of their health during pregnancy to avoid any potential risk factors. Pregnancy itself imposes different physiological and behavioral changes in the pregnant woman and different factors influence the pregnancy conditions and outcomes. The present study aims to evaluate the reasons and causes of headache among pregnant women attending the emergency department (Imam Khomeini Hospital, Sari).

**Methods:** This was a descriptive cross-sectional study conducted 30 pregnant women attending the emergency department because of headache during October to December 2011. Sampling method was counting all patients.The patients were assessed at the end of their treatment process using a customized questionnaire. All patients attended the department for treatment during the study period voluntarily answered the items of the questionnaire.

**Results:** A total of 30 pregnant women complaining about persistent headaches attended the emergency department. They were assessed for the pregnancy poisoning. 19 patients experienced headache during the third trimester of pregnancy. Assay poison of pregnancy women &19 women stricken headaches in three month last of pregnancy.

**Conclusions:** If the headaches of pregnant women in their third trimester of pregnancy are accompanied by hypertension and proteinuria, they should visit a specialist to assess the presence of pre-eclampsia. When the pre-eclampsia test is positive, the patient should be seriously treated. Proteinuria test was performed on all patients and all of them have hypertension. In a rare condition the hypertension and proteinuria occur in first days of post-delivery. The cause of high blood pressure may be the pregnancy itself which is transient, or the patients had hypertension prior to the pregnancy and not aware of their problem. In the latter case, the problem can persist after pregnancy. Long-time hypertension increases the risk of pre-eclampsia so that 25% women, who have hypertension, experienced pre-eclampsia, while only 5% pregnant women usually experience pre-eclampsia. Therefore, if the blood pressure is high in a pregnant woman without any previous history, it cannot be predicted that the hypertension either remains unchanged, increases or leads to pre-eclampsia. If the patient catches pre-eclampsia, the proteinuria test shows excess level of urine protein that leads to swelling in the arms and legs. This symptom indicates the failure of kidney function as a result of hypertension. In some cases both the mother and fetus are under serious risks. Pre-eclampsia and hypertension are among serious health threats for pregnant women and also the most common cause of mortality in pregnant women. Therefore, presence of headache in the third trimester of pregnancy should be treated seriously.

### Keywords:

***Pregnancy, Pre-eclampsia, Headache, Third trimester, Proteinuria***

* ***Corresponding Author at:***
***Seyyed Hossein Montazer:*** *Associate Professor of Department Emergency Medicine, Mazandran University of Medical Science, Mazandaran, Iran, Email:hmontazer66@yahoo.com,* ***(Montazer SH.).***

